# Smartphone-Enhanced Symptom Management In Psychosis: Open, Randomized Controlled Trial

**DOI:** 10.2196/17019

**Published:** 2020-08-13

**Authors:** Shon Lewis, John Ainsworth, Caroline Sanders, Charlotte Stockton-Powdrell, Matthew Machin, Pauline Whelan, Richard Hopkins, Zhimin He, Eve Applegate, Richard Drake, Charlie Bamford, Chris Roberts, Til Wykes

**Affiliations:** 1 Manchester Academic Health Sciences Centre Greater Manchester Mental Health Foundation Trust The University of Manchester Manchester United Kingdom; 2 Institute of Psychiatry, Psychology and Neurosciences King's College London London United Kingdom; 3 South London and Maudsley NHS Foundation Trust London United Kingdom

**Keywords:** digital, smartphone, m-health, psychosis, mental health, self management

## Abstract

**Background:**

Improving recovery from acute symptoms and preventing relapse are two significant challenges in severe mental illness. We developed a personalized smartphone-based app to monitor symptoms in real time and validated its acceptance, reliability, and validity.

**Objective:**

To assess (i) acceptability of continuous monitoring to SMI patients and health professionals over 3 months; (ii) impact of active self-monitoring on positive psychotic symptoms assessed at 6 and 12 weeks; and (iii) the feasibility of detecting early warning signs of relapse.

**Methods:**

The active symptom monitoring smartphone app was built into an end-to-end system in two NHS Trusts to enable real-time symptom self-monitoring and detection by the clinical team of early signs of relapse in people with severe mental illness. We conducted an open randomized controlled trial of active symptom monitoring compared to usual management to assess: (i) acceptability and safety of continuous monitoring over 3 months; (ii) impact of active self-monitoring on positive psychotic symptoms assessed at 6 and 12 weeks; (iii) feasibility of detecting early warning signs of relapse communicated to the healthcare staff via an app streaming data to the electronic health record. Eligible participants with a Diagnostic and Statistical Manual of Mental Disorders, 4th Edition (DSM-IV) diagnosis of schizophrenia and related disorders, and a history of relapse within the previous two years were enrolled from an early intervention team and a community mental health team.

**Results:**

Of 181 eligible patients, 81 (45%) consented and were randomized to either active symptom monitoring or management as usual. At 12 weeks, 90% (33/36) of those in the active monitoring group continued to use the system and exhibited an adherence rate (defined as responding to >33% of alerts) of 84% (30/36}. Active symptom monitoring was associated with no difference on the empowerment scale in comparison to the usual management group at 12 weeks. The pre-planned intent-to-treat analysis of the primary outcome, a positive score on the Positive and Negative Syndrome Scale (PANSS) scale, showed a significant reduction in the active symptom monitoring group over 12 weeks in the early intervention center. Alerts for personalized early warning signs of relapse were built into the workflows of both NHS Trusts, and 100% of health professional staff used the system in a new digital workflow. Qualitative analyses supported the acceptability of the system to participants and staff.

**Conclusions:**

The active smartphone monitoring system is feasible and was accepted by users in a 3-month study of people with severe mental illness, with surprisingly high levels of adherence. App use was associated with psychotic symptom improvement in recent-onset participants, but not those with longstanding illness, supporting the notion of improved self-management. When built into clinical management workflows to enable personalized alerts of symptom deterioration, the app has demonstrated utility in promoting earlier intervention for relapse.

**Trial Registration:**

ISRCTN Registry ISRCTN88145142; http://www.isrctn.com/ISRCTN88145142

## Introduction

Severe mental illnesses such as schizophrenia often run a relapsing, lifelong course. Persons with severe mental illness have two primary goals: to improve the speed and quality of their recovery and to prevent future relapse. Following the first episode, 70% will have at least one relapse during the next five years [1]. Despite the rise of community care, 40% of the costs of care for a person with severe mental illness are on unplanned inpatient care for relapse [2]. In standard UK practice, contact with health professionals typically occurs only once every 2-6 weeks, so that early signs of relapse are usually picked up too late to enable prompt intervention. Early warning signs of relapse usually comprise the emergence of a mixture of dysphoric symptoms such as anxious mood, and then attenuated psychotic symptoms, appearing over 1-5 days, with insight usually retained until the day of relapse [3].

We developed a smartphone-based platform in 2010 (ClinTouch) to help persons with severe mental illness to manage their symptoms and prevent relapse. Randomized feasibility trials showed this method of active symptom monitoring to be safe, feasible, and acceptable to people with severe mental illness [4,5]. Users with severe mental illness preferred the smartphone app to an equivalent SMS-based version, which took longer to complete (mean 326 seconds versus 68 for the smartphone app [6]).

Having demonstrated the proof of concept, we integrated the standalone smartphone system (ClinTouch) via an application programming interface (API) into NHS Trust information and communication technology platforms. This integration enabled the streaming of summary information into electronic health records, enabling health professionals to track current symptoms on desktops at the team base and receive personalized alerts when symptoms exceeded a pre-agreed threshold.

This report describes an open randomized controlled trial of smartphone-based active symptom management versus usual care to assess the (i) acceptability and safety of continuous monitoring in persons with severe mental illness and health professionals over 3 months, (ii) impact of active self-monitoring on positive psychotic symptoms assessed at 6 and 12 weeks, and (iii) feasibility of detecting early warning signs of relapse.

## Methods

### Study Design

The trial of ClinTouch active symptom management versus management as usual was a two-center, open, randomized controlled trial at the NHS Mental Health Trusts in Manchester and South London. Software development, beta testing, and prior cohort and smaller randomized trials had used an experience-driven design process in which service users with severe mental illness were involved in all stages of the design and development of the app, its functionality, and its standard operating procedures. Health professionals were included in design issues where they related to the use of the system within routine practice and in the design of new, digitally-enabled workflows. In preparing for the current trial, 6 focus groups were conducted and audiotaped, including a total of 23 service users, 5 carers, and 30 healthcare staff. Qualitative in-depth interviews were conducted with 19 service users, 6 carers, and 17 staff. A Service User and Carer advisory group met quarterly throughout the project and provided advice on study design, information for participants, and related issues.

The personalized smartphone app triggers the user to rate their symptoms several times a day, and wirelessly uploads these in real time to a secure central server. An audio cue triggered semi-randomly 2-4 times a day reminds the user to complete a set of 12-14 branching items about current symptom severity using a touchscreen slider. A graphical summary of how symptoms fluctuate over time is assembled and displayed on the handset. By conducting face-to-face interview assessments using the gold standard Positive and Negative Syndrome Scale (PANSS) [7] before and after one week of 4 times daily ClinTouch assessment, we confirmed the validity of the self-reported items. Core psychotic symptom and mood items showed moderate to strong (r>0.6) correlations between the in-person and self-report methods [5]. Non-core, behaviorally assessed items such as negative symptoms showed weaker correlations.

The trial was approved by the South Birmingham NHS Research Ethics Committee (14/WM/0045). The trial was registered with the National Institute of Health Research CRN portfolio: 16361, and ISRCTN 88145142. The Medicines and Healthcare Regulatory Agency elected not to designate ClinTouch as a medical device as deployed for the trial.

### Participants

One community clinical team from each Trust participated. In England, community mental health teams serve a geographically defined catchment area. All Trusts use electronic patient record systems. Management of individual service users is coordinated by a mandatory care coordinator, usually with a nursing background. Each team has one consultant psychiatrist working with psychologists and other mental health professionals. Teams typically have caseloads of 200-400 people with severe mental illness, with 20-30 care coordinators. In the South London Trust, an Early Intervention for Psychosis (EIP) team was selected and in the Manchester Trust, a Community Mental Health Team (CMHT). NHS EIP teams are configured to manage care for people in the first three years after the first psychotic episode, after which care is transferred to a CMHT. Different types of teams were chosen a priori to investigate the effect of duration of illness on any response to digital treatment. Recruitment took place between February 2014 and May 2015.

Participant inclusion criteria were: (i) operational Diagnostic and Statistical Manual 8^th^ Edition DSM-IV [8] diagnosis of schizophrenia and related disorders; (ii) aged 16-65; (iii) one or more psychotic episodes in the previous 2 years, including the first psychotic episode. Exclusion criteria were: (i) unable to speak English; (ii) unable to give informed consent. Patients who met these criteria were identified separately in the two clinical teams.

### Randomization

Participants were allocated by computer using randomized, permuted blocks to one of two groups: active symptom monitoring plus management as usual, or management as usual alone, each for 12 weeks. No stratification was used.

### Procedure/Intervention

There were two linked interventions. Active symptom monitoring with feedback to participants was aimed at encouraging self-management of symptoms. Alerts were fed back to the care coordinator when personalized early warning sign thresholds were exceeded, allowing very early intervention.

The ClinTouch active symptom management system was integrated into the electronic health record (EHR) platform of the Manchester NHS Trust via an application programming interface (API). The API instructed the EHR to retrieve data from the ClinTouch dashboard, including a list of participants using the ClinTouch system and any alerts associated with them. The EHR system displayed information relating to the ClinTouch data within the individual patient record. Care coordinators and clinicians were given secure individual logins to the ClinTouch system, enabling them to view the data on a desktop along with graphs of symptom changes over time. The EHR provider for the South London Trust denied API access. To compensate for this lack of access, an automated email was sent to the appropriate care coordinator whenever a patient alert was raised. The email solution was also employed in the Manchester Trust.

Frontline clinical staff (n=42) were trained in the use of ClinTouch. For patients randomized to the experimental group, the relevant care coordinator delivered training in the use of the handset. Either the Android app was installed on the participant’s phone, or a preconfigured Samsung Galaxy smartphone was provided on loan for the duration of the study. The branching items covered positive psychotic symptoms, anxiety, and mood as validated against the PANSS scale in previous studies. Semi-random twice-daily auditory cues from the handset prompted symptom data collection and wireless upload. The system was then used for 12 weeks in the context of the preexisting care plan modified for the ClinTouch algorithms.

Care coordinators then determined the criteria for each participant’s early warning signs, using previous electronic patient records for reference. The early warning sign threshold was set by assigning a score of 0-3 (low through high) to each symptom according to how relevant it is for that participant’s relapse signature based on previous experience. The alert algorithm was constructed so that symptoms scored as 1 collectively comprised 20% of the total early warning sign score, those scoring 2 comprised 30%, and those scoring 3 comprised 50%. An alert was generated if the total score for a single datapoint rose to 40% higher than the baseline defined as the mean score for the first 3 days of recording, or 25% higher across two consecutive data points. Operationally, this was done as part of the standard crisis planning meeting.

Standard operating procedures were established for recording and handling adverse events. Technical measures to ensure data privacy and patient confidentiality followed industry-standard best practices, and all data communications between app and server used encrypted channels. Data were handled per the UK Data Protection Act 1998.

### Follow-up and Assessments

Participants were assessed in an in-person interview at baseline, then 6 and 12 weeks after randomization. Research assistants trained to criterion inter-rater reliability undertook participant assessments.

Feasibility and acceptability outcomes in the experimental group were two-fold. The client-centered outcomes included the proportion of eligible clients consenting to a trial of ClinTouch active symptom management. We predicted that 50% would remain in follow-up for 12 weeks. We predicted that 50% of participants would complete >33% of all possible symptom self-ratings over the 12-week trial. The clinical team outcomes included the proportion of all care coordinators accessing patients’ online symptom data. Adverse effects were routinely monitored during the weekly telephone support calls to participants.

Primary efficacy endpoints over 12 weeks included (i) Score on the positive symptom subscale of the PANSS, (ii) user empowerment from interviews, and the Empowerment Rating Scale [9]. Secondary efficacy outcomes were (i) Calgary Depression Scale [10], (ii) Global Assessment of Functioning scale (GAF) [8], and (iii) health-related quality of life, the EuroQol 5D (EQ5D) [11]. These face-to-face interviews were recorded in hard copy versions of the rating scales and the data stored securely in accord with Medical Research Council guidance.

In order to gain an estimate of how frequently clinical staff recorded episodes of possible early warning signs independently of the active symptom monitoring system, transcripts of electronic care records for the 12 weeks of the trial plus a further 4 weeks were anonymized and any reference to randomized treatment redacted. These were then rated independently by two experienced clinicians (SL, RH) for the documented occurrence of emergent symptoms, which met early warning criteria of documented worsening of psychotic symptoms.

Qualitative interviews were conducted in a subsample of those declining to participate and those allocated to ClinTouch at exit.

### Statistical Analysis

The effect of ClinTouch-enhanced monitoring on PANSS Positive Subscale totals at follow-up was examined using analysis of covariance (ANCOVA), including allocation group and site (Manchester or London) as cofactors and baseline scores as a covariate, using Stata 14.1 (College Station). The teams at each site were selected purposely so that differences in response between young, recent onset participants (London) and older, more chronically unwell participants (Manchester) could be examined. Sensitivity analyses examined the effect of demographic variables (covariates were sex, age, level of qualifications, ethnic minority status, living independently, being single, unemployed, in current psychotherapy or abusing alcohol) using backward stepwise elimination of associations of *P*>.20. A comparison of individual general linear models for the two sites was pre-planned to examine the likely differences. Finally, secondary analyses of other PANSS subtotals and total were conducted in the same way as the primary analysis.

The sample size was calculated based on a 50% reduction in early warning signs in the experimental treatment arm over 12 weeks, from 40% to 20%. Assuming a 10% drop out rate, a sample size of 72 would have 80% power to detect this difference with a one-sided alpha of 0.2, as recommended for a feasibility trial. The analysis was by intent to treat ANCOVA using STATA, with data at baseline, then 6 and 12 weeks.

## Results

### Recruitment and Feasibility

**Figure 1:**
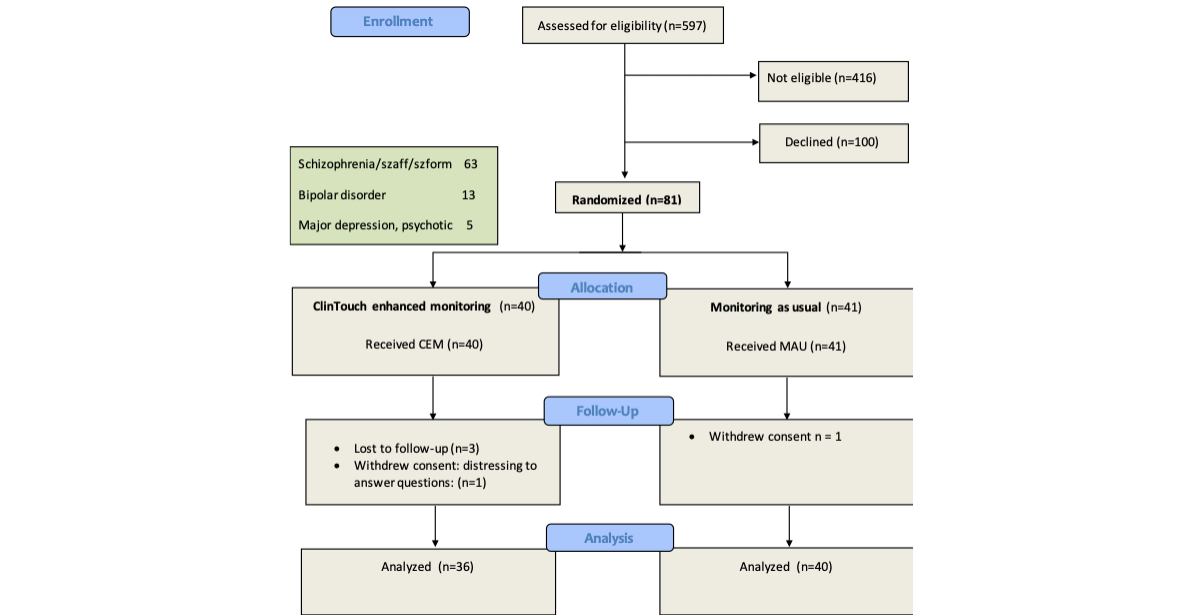
Randomized controlled trial flow diagram.

Of 181 eligible service users approached, 81 (46%) consented to participate and were randomized to either ClinTouch-enhanced management or management as usual (see Tables 1 and 2, Figure 1). There were substantial demographic differences between sites (see Table 2), as intended and expected. The CMHT participants (Manchester) were older and chronically unwell (mean 46 years; median 2.5 hospital admissions, IQR 1 to 4) than the EIT participants (mean 26 years; London: median 1 admission, IQR 0 to 1). Of those 40 who were randomized to the ClinTouch-enhanced management arm, 38 (95%) stayed in the trial for 12 weeks. Of these 38, acceptable adherence as defined by responding to at least 33% of beep alerts (four-item sets per day) was 84%, good adherence (greater than 50% of alerts) was 60%. Healthcare professionals (care coordinators) used ClinTouch-enhanced management in 100% of cases, accessing ClinTouch data an average of 24 times per patient.

**Table 1:** Demographic data by treatment group.

Descriptor	ClinTouch enhanced monitoring plus standard care	Standard care
Number	40	41
Age (years), mean (range)	33.7 (21-61)	35.3 (20-68)
Sex - female	11	16
Ethnicity - white	20	23
Ethnicity - black/black British	17	15
Ethnicity - other	3	3

**Table 2:** Demographic data by site.

Descriptor	Community mental health (Manchester)	Early psychosis (London)
Number	37	44
Active symptom monitoring treatment arm	18	22
Age (years), mean (range)	46.1 (21-68)	26.1 (19-36)
Sex - female	14	13
Ethnicity - white	31	12
Ethnicity - black/black British	6	26
Ethnicity - other	0	6

### Safety

Adverse effects were routinely monitored during weekly telephone support calls. Of 38 participants who completed 12 weeks of the trial, three (8%) reported significant events: 1 reported increased anxiety prompted by questions; 1 reported increased irritation due to the alert beeps, and 1 had their charger explode. All 3 continued to complete the 12 weeks in the trial.

### Clinical efficacy

There were no substantial differences in symptom severity at the point of randomization between those allocated to ClinTouch-enhanced monitoring or standard care (Table 3). On the primary efficacy outcomes, there was no significant difference between groups in PANSS Positive total after 6 or 12 weeks, nor were there significant differences in secondary outcomes (Table 3). Sensitivity analysis showed that including demographic variables made no substantial difference to the allocation group’s coefficient or significance.

The planned analyses of each site separately demonstrated different outcomes in the different services. Although there were no significant differences between ClinTouch-enhanced monitoring and control participants in Manchester (apart from a difference in depression scores identifiable at baseline and persisting without significant alteration during the trial; Table 4), findings in London were different (Table 5). There was a significant reduction in positive symptoms after 12 weeks of ClinTouch-enhanced monitoring in the early psychosis subsample (adjusted mean difference –3.04; CI –5.49, –0.59; *P*=.016. Although there was a significant site-by-group interaction for PANSS total (Supplementary Table 1; *P*=.003), indicating a significantly lower PANSS total after 12 weeks of ClinTouch-enhanced monitoring in the early psychosis center, this benefit was not in itself significant (adjusted mean difference –5.83; CI –14.14, 2.48; *P*=.164 2-tailed). There were no other significant site-by-group differences. In addition to the conventional rating scales, the ClinTouch device provided real-time individual active symptom data, which indicated that over 12 weeks, all symptoms except one declined in mean severity. Severity of hallucinations decreased by 29%.

The frequency of early warning signs, as documented in electronic patient records, was 33% in the CEM group and 46% in the control group over 12 weeks, after excluding 8 cases where records were too scant to be rated. The actual performance of this early prototype of the Early Warning Signs algorithm was suboptimal, in terms of the accuracy of ClinTouch alerts versus early warning signs as contemporaneously documented in the electronic patient record. Sensitivity was 75%, specificity 8%, giving a positive predictive value of 29%.

**Table 3:** Clinical Measures at baseline, 6, and 12 weeks by allocation group.

Scale and visit		CareLoop enhanced monitoring, mean (SD)	Management as Usual, mean (SD)	Adjusted mean difference^a^	95% CI	*P* value	*P* value	Intercept site*trial arm *P*
						2-tailed	1-tailed	
**PANSS^b^** **Total**								
	Baseline	72.9 (14.8)	76.8 (17.4)					
	Weeks 6	70.7 (17.0)	73.9 (20.7)	–0.47	–6.47 to 5.53	.874	.44	.46
	Week 12	64.5 (15.7)	69.3 (20.7)	–1.93	–7.50 to 3.64	.492	.25	.003
**PANSS Positive**								
	Baseline	18.8 (5.4)	18.3 (5.7)					
	Weeks 6	17.3 (6.2)	17 (6.2)	–0.37	–2.35 to 1.60	.708	.35	.34
	Week 12	16 (5.3)	16.7 (6.2)	–1.13	–3.12 to .87	.264	.13	.057
**PANSS Negative**								
	Baseline	18.8 (4.3)	18.3 (5.5)					
	Weeks 6	16.1 (4.4)	18.2 (5.7)	–0.44	–2.18 to 1.30	.616	.31	.75
	Week 12	15 (4.4)	17.1 (5.6)	–0.69	–2.51 to 1.15	.462	.23	.53
**PANSS General**								
	Baseline	38.2 (8.7)	40 (9.2)					
	Weeks 6	37.4 (9.5)	38.7 (10.9)	–0.17	–3.70 to 3.37	.994	.47	.64
	Week 12	33.5 (8.6)	35.5 (10.7)	–0.79	–3.86 to 2.29	.611	.31	.38
**ERS^c^** **Total**								
	Baseline	86.3 (7.4)	81.4 (7.8)					
	Weeks 6	85.4 (7.6)	81.6 (10.3)	0.58	–2.99 to 4.15	.748	.37	.47
	Week 12	86.5 (11.9)	83.6 (8.1)	–0.05	–4.35 to 4.25	.983	.49	.32
**EQ5D^d^** **Total**								
	Baseline	8.8 (3.1)	9.6 (4.1)					
	Weeks 6	9.2 (3.4)	8.8 (4.2)	–0.29	–2.43 to 1.85	.286	.14	.29
	Week 12	8.0 to 4.1	8.4 (3.8)	0.15	–1.23 to 1.53	.812	.41	.15
**CDS^e^** **Total**								
	Baseline	5.8 to 4.6	8.1 (5.6)					
	Weeks 6	6 to 4.5	7.3 (5.2)	0.29	–1.30 to 1.90	.712	.36	.03
	Week 12	4.6 to 3.7	6.5 (4.8)	–0.67	–2.24 to 0.90	.4	.20	.83
**GAF^f^**								
	Baseline	49.7 to 14.9	49.3 (11.8)					
	Weeks 6	49.2 to 14.5	47.7 (16.7)	–0.62	–6.45 to 5.21	.595	.23	.90
	Week 12	51.8 to 13.7	52.2 (16.2)	–2.65	–8.38 to 3.07	.850	.43	.30

^a^Follow-up differences adjusted for baseline scores and the main effect of site.

^b^PANSS: Positive and Negative Syndrome Scale

^c^ERS: Empowerment Rating Scale

^d^EQ5D: EuroQol-5D

^e^CDS: Calgary Depression Scale

^f^GAF: Global Assessment of Functioning

**Table 4:** Clinical measures at baseline, 6 weeks, and 12 weeks: community team sample.

Scale and visit		CareLoop enhanced monitoring, mean (SD)	Management as usual, mean (SD)	Adjusted mean difference^a^	95% CI	*P* value, 2-tailed
**PANSS^b^** **Total**						
	Baseline	72.73 (11.71)	78.32 (19.02)			
	Weeks 6	69.47 (17.43)	72.68 (22.53)	1.30	–8.98 to 11.58	.80
	Week 12	57.84 (14.23)	68.59 (22.22)	–5.83	–14.14 to 2.48	.16
**PANSS Positive**						
	Baseline	19 (4.22)	19.36 (6.12)			
	Weeks 6	16.42 (5.67)	17.41 (6.65)	–0.95	–3.91 to 2.01	.52
	Week 12	14.11 (4.10)	17.18 (6.27)	–3.04	–5.49 to –0.59	.02
**PANSS Negative**						
	Baseline	16.09 (4.21)	18.77 (5.52)			
	Weeks 6	15.42 (4.50)	17.32 (6.12)	0.004	–2.83 to 2.92	.98
	Week 12	13.47 (4.58)	16.45 (6.10)	–0.76	–3.36 to 1.85	.56
**PANSS General**						
	Baseline	37.64 (7.49)	40.18 (10.13)			
	Weeks 6	37.63 (10.12)	37.95 (11.46)	1.43	–4.51 to 7.36	.63
	Week 12	30.26 (8.55)	34.95 (11.15)	–2.67	–7.46 to 2.13	.27
**ERS^c^** **Total**						
	Baseline	86.73 (5.59)	83.27 (6.53)			
	Weeks 6	85.74 (5.69)	83.68 (8.16)	0.13	–4.18 to 4.43	.95
	Week 12	86.26 (6.15)	85.91 (8.07)	–1.26	–5.80 to 3.29	.58
**EQ5D^d^** **Total**						
	Baseline	8.09 (2.81)	8.64 (3.09)			
	Weeks 6	6.91 (3.53)	8.23 (3.16)	1.00	–0.78 to 2.79	.26
	Week 12	6.45 (3.49)	7.00 (2.31)	0.33	-1.35 to 0.83	.20
**CDS^e^** **Total**						
	Baseline	5.82 (4.16)	8.5 (5.86)			
	Weeks 6	6.63 (4.21)	6.73 (4.46)	1.60	–0.486 to 3.67	.13
	Week 12	4.21 (3.03)	6.27 (4.38)	–0.85	–2.94 to 1.25	.42
**GAF^f^**						
	Baseline	42.38 (12.04)	38.4 (6.42)			
	Weeks 6	44.47 (13.29)	41.91 (16.25)	–0.11	–9.40 to 9.17	.98
	Week 12	48.47 (11.75)	45.14 (18.93)	–1.09	–10.05 to 7.00	.80

^a^Follow-up differences adjusted for baseline scores.

^b^PANSS: Positive and Negative Syndrome Scale

^c^ERS: Empowerment Rating Scale

^d^EQ5D: EuroQol-5D

^e^CDS: Calgary Depression Scale

^f^GAF: Global Assessment of Functioning

**Table 5:** Clinical measures at baseline, 6, and 12 weeks: First episode psychosis sample.

Scale and visit		CareLoop enhanced monitoring, mean (SD)	Management as usual, mean (SD)	Adjusted mean difference^a^	95% CI	*P* value, 2 tailed
**PANSS^b^** **Total**						
	Baseline	73.11 (18.16)	75.00 (15.72)			
	Weeks 6	72.06 (16.86)	75.44 (18.77)	–2.84	–8.81 to 3.12	.34
	Week 12	71.50 (14.31)	70.11 (19.24)	2.69	–4.81 to 10.19	.47
**PANSS Positive**						
	Baseline	18.44 (6.66)	17.16 (5.1)			
	Weeks 6	18.24 (6.82)	16.56 (5.79)	2.67	–2.48 to 3.01	.84
	Week 12	18.06 (5.86)	16.06 (6.35)	1.10	–2.16 to 4.35	.498
**PANSS Negative**						
	Baseline	15.83 (4.48)	17.95 (5.69)			
	Weeks 6	16.76 (4.28)	19.28 (5.04)	–1.03	–3.01 to .94	.30
	Week 12	16.5 (3.73)	18.06 (4.83)	–0.50	–3.09 to 2.10	.70
**PANSS General**						
	Baseline	38.83 (10.17)	39.84 (8.34)			
	Weeks 6	37.06 (9.13)	39.61 (10.32)	–2.32	–5.85 to 1.23	.19
	Week 12	36.94 (8.91)	36.11 (10.44)	1.33	–2.60 to 5.26	.495
**ERS^c^** **Total**						
	Baseline	85.83 (9.32)	79.26 (8.84)			
	Weeks 6	85 (9.52)	78.94 (12.25)	1.40	–4.73 to 7.53	.65
	Week 12	86.72 (16.05)	80.72 (7.43)	1.63	–6.25 to 9.50	.68
**EQ5D^d^** **Total**						
	Baseline	9.5 (3.59)	11.21 (4.60)			
	Weeks 6	9.22 (4.25)	10.42 (4.86)	0.10	–2.48 to 2.69	.94
	Week 12	8.06 (7.38)	6.116 (5.17)	–1.40	–5.72 to 2.02	.51
**CDS^e^** **Total**						
	Baseline	5.72 (5.21)	7.68 (5.23)			
	Weeks 6	5.29 (4.81)	8.06 (6.00)	–1.36	–0.98 to 3.69	.25
	Week 12	5.06 (4.41)	6.83 (5.48)	–0.63	–1.81 to 3.07	.61
**GAF^f^**						
	Baseline	56.94 (13.12)	53.84 (12.30)			
	Weeks 6	52.64 (14.52)	51.56 (16.79)	–1.19	–8.51 to 6.15	.74
	Week 12	54.11 (14.65)	57.17 (10.78)	–5.09	–12.38 to 2.20	.17

^a^Follow-up differences adjusted for baseline scores.

^b^PANSS: Positive and Negative Syndrome Scale

^c^ERS: Empowerment Rating Scale

^d^EQ5D: EuroQol-5D

^e^CDS: Calgary Depression Scale

^f^GAF: Global Assessment of Functioning

## Discussion

We conducted an open randomized controlled trial of active symptom monitoring compared to usual management in people with serious mental illness to assess over 12 weeks the (i) acceptability and safety of continuous monitoring, (ii) impact of active self-monitoring on positive psychotic symptoms, and (iii) feasibility of detecting early warning signs of relapse communicated to the healthcare staff via an API allowing data to be streamed into the EHR.

A systematic review has suggested that smartphone apps may be helpful in the management of mental health disorders such as depression [12]. Nonetheless, almost none of the publicly available mental health apps have good quality data concerning safety and efficacy [13]. The real-time digital approach used in this study holds several advantages over routine clinical assessment. It reduces the confounding effects of retrospective recall bias, forgetting, and averaging in symptom appraisal. It allows the context of symptom changes to be assessed and increases patient involvement in continuing care through participation in symptom and progress monitoring. It may also enable a degree of symptom self-management via a trusted and ubiquitous, ever-present personal device.

The trial demonstrated several things. The active symptom monitoring intervention was safe and acceptable: 45% of the eligible sample agreed to enter the trial. Furthermore, and importantly, of those using the ClinTouch-enhanced monitoring system, 90% continued to use it regularly at 3 months. In these patients, adequate adherence was 84%, defined as responding to >33% of item prompts. On pre-planned intent-to-treat analysis, the primary outcome of positive symptom score on the PANSS scale showed a significant reduction in the ClinTouch group over 12 weeks only in the early intervention center. The larger therapeutic effect in the early psychosis participants was not due to the severity or adherence differences between the two subsamples. It may be that, as has been shown with pharmacological and psychological treatments for psychosis, the therapeutic effect is larger earlier in the course of the disorder.

We have demonstrated from a software perspective that we can build an algorithm into the ClinTouch app to provide an alert when symptoms start to worsen. An API allowed this to be built into the electronic patient record system in one Trust. With symptom data streamed into the EHR system, health professionals could view it on a secure desktop at the team base. Alerts for early warning signs were built into the workflows of the two NHS Trusts, and 100% of health professional staff used the system to access symptom data and alerts in a new digital workflow. Qualitative analyses supported the acceptability of the system to participants and staff.

There were limitations to the trial. In the second Trust, the commercial provider of the EHR did not comply with the study, indicating a potential barrier to full scale roll out in the NHS where Trusts have a range of different commercially provided EHR platforms. Another limitation was that, at the time of the trial (2014-2016), the ClinTouch app was only available for the Android operating system. In addition, the accuracy of the early prototype in detecting EWS was limited by our focus being mainly on operability. Case record documentation of EWS was often scanty, proving to be an inadequate gold standard. Artifacts in functionality were identified for improvement, such as alerts being mistimed if the user was temporarily in an area without a wireless network. Subsequent versions are proving more refined. Further work is now taking place to refine the alert algorithm through robust risk prediction modeling in order to increase its sensitivity and specificity and improve the effectiveness of promoting early intervention by clinical teams to improve patient outcomes.

In conclusion, the active smartphone monitoring system is feasible and acceptable over three months to users with severe mental illness, with surprisingly high levels of adherence both from users and health professionals. It was associated with psychotic symptom improvement in patients with recent-onset psychosis, and supports the notion of improved self-management in those with first episode psychosis. In terms of implications for clinical practice, digital health interventions appear to hold considerable promise in the management of people with psychosis. Smartphone-based active symptom monitoring can be built into EHR systems and regular clinical workflows and allow preventive, personalized care, especially if combined in with added digital functionality such as medication management and physical health monitoring.

## Appendix

Multimedia Appendix 1 CONSORT eHEALTH checklist (V 1.6.1).

## Abbreviations

ANCOVA: analysis of covariance
API: application programming interface
CDS: Calgary Depression Scale
CMHT: Community Mental Health Team
DSM-IV: Diagnostic and Statistical Manual of Mental Disorders, 4th Edition
EHR: electronic health record
EIP: Early Intervention for Psychosis
EQ5D: EuroQol 5D
GAF: Global Assessment of Functioning
PANSS: positive and negative syndrome scale
